# Assessment of Emerging Technologies to Support Individuals With At-Risk Alcohol Consumption: Pilot Controlled Investigation Study

**DOI:** 10.2196/83592

**Published:** 2026-03-06

**Authors:** Karl Andersson, Linda Handlin, Sanela Huskic Beslic, Rajna Knez, Afrouz Behboudi, Marie Wilhsson

**Affiliations:** 1 Skillsta Teknik Design och Kvalitet AB, Vänge, Sweden; 2 DHEAR, University of Skövde, Högskolevägen, Skövde, 54128, Sweden, 46 500448000; 3 Department of Psychiatry, Skaraborg Hospital, Skövde, Sweden

**Keywords:** mobile health, alcohol, biomarkers, digital health, eye scanning

## Abstract

**Background:**

The Swedish National Board of Health and Welfare recently updated the national guidelines for at-risk consumption of alcohol. Nearly 30% of the Swedish population now falls under the at-risk category and should be provided with support.

**Objective:**

This project aims to identify and evaluate efficient, scalable tools to support individuals with risk-prone alcohol consumption. The project seeks to explore innovative, accessible technologies that could be implemented in large-scale public health interventions.

**Methods:**

A pilot-scale clinical study was conducted to assess the feasibility of using emerging technologies for this purpose. Eight healthy volunteers participated in controlled alcohol consumption while being monitored through 2 methods: an eye-scanning tool integrated into a standard mobile phone and saliva sampling for biomarkers such as serotonin and orexin.

**Results:**

Eye-scanning parameters began to shift in some participants at approximately 0.4 to 0.5 per mille blood alcohol concentration, particularly in the form of impaired eye convergence. Furthermore, at around 0.5 per mille, participants encountered practical difficulties in managing the eye-scanning app. Salivary biomarkers did not show any clear correlation with alcohol intake, presumably due to the low number of participants. Beyond biological findings, the study also generated important procedural insights for designing a large-scale clinical study.

**Conclusions:**

Eye scanning showed potential as a noninvasive and accessible method for detecting and monitoring moderate alcohol consumption effects, while serotonin and orexin biomarkers were not informative in this context. On the basis of these findings and procedural learnings, eye-scanning tools warrant further investigation in larger clinical studies aimed at developing scalable support for risk-prone alcohol consumption.

## Introduction

The Swedish National Board of Health and Welfare has changed the limit for risk-prone alcohol consumption and proposes that all individuals at risk shall be offered support [1]. This is a daunting task, as according to a report from the Swedish Council for Information on Alcohol and Other Drugs published in February 2024, risky alcohol consumption was estimated at 31% across all age groups, except for those aged 50 to 64 years, where the proportion was 35%. Furthermore, the prevalence of risky use was higher (36%) in urban-adjacent areas compared to smaller towns and rural areas (30%) [2]. Hazardous alcohol consumption among Swedes aged 16 to 84 years is around 15%, even before the updated definitions of harmful drinking. According to the Public Health Agency of Sweden, hazardous alcohol consumption is more prevalent in young people and men [3]. Taken together, these figures highlight the magnitude and heterogeneity of the target population, underscoring the substantial demand on health services. To adequately respond to the needs of this sizable and diverse population, all support systems must be structured with a clear focus on both efficiency and scalability. In addition, the population needs to be informed of the hazards of moderate drinking to motivate acceptance of support.

Continuous monitoring of risk factors to maintain public health is common. Most programs screen the population at risk; for example, mammography screening for breast cancer typically begins for individuals aged 40 to 50 years. An increasing number of biomarker-driven screening tests are discussed, including those targeting specific diseases [4], those targeting generic longevity through multipanel approaches [5], and practical elements such as patient-participation considerations [6]. With alcohol being a significant contributor to illness [7], it is logical that monitoring programs for alcohol consumption should be implemented. Several possible interventions have been evaluated [8]. However, the social role of alcohol in everyday life probably makes policymakers hesitant.

Smartphone-based support tools for alcohol addiction therapy have been available for many years. One of the more advanced support tools allows frequent testing of breath alcohol using a breathalyzer in combination with tools for motivational interviewing (MI) and cognitive behavioral therapy (CBT), where the system is capable of even predicting relapses ahead of time [9]. Breathalyzers that assess breath alcohol concentration are particularly useful for delivering real-time information to both individuals receiving care and health care providers. This intervention represents an effective and proactive method for monitoring treatment [10]. MI is a collaborative, person-centered counseling approach that addresses ambivalence and enhances motivation for change [11]. It is widely used in health care and behavioral interventions to support adoption and maintenance of healthier behaviors. CBT has progressed through 3 waves: the first focused on behavioral principles; the second on cognitive processes; and the third on acceptance, mindfulness, and values. MI and CBT are 2 well-established, evidence-based psychotherapies that have been shown, particularly in combination, to be effective for conditions including anxiety, depression, and substance abuse [12].

We hypothesize that the support scenario concept for at-risk consumers could include 1 branch of support and 1 branch of testing. The support branch aims to reduce the alcohol consumption, and the test branch aims to monitor whether an individual is still within, or has left, the risk-consumption category. Using the already deployed support system for the management of alcohol use disorder as a template, a support system based on MI and CBT is feasible. However, self-reported consumption is not considered an option, due to underreporting [13], and testing a large population for alcohol consumption using a breathalyzer is very costly, indicating that other feasible and cost-effective approaches are necessary. Wearable sensors allowing direct measurements of alcohol are emerging [12] but are still not ready for large-scale deployment. In this context, measurements of secondary markers, that is, metabolites or bodily reactions, can provide the scale necessary for the intended population.

Individuals with elevated alcohol consumption also face increased risk of mental health issues such as anxiety, altered stress sensitivity, depression, and aggressive behavior. These conditions are often linked to changes in neurotransmitter and neurohormonal systems, including serotonin, oxytocin, orexin, and dopamine [14,15]. Women of childbearing age are also at risk for unknowingly affecting the offspring, where the prevalence of fetal alcohol spectrum disorders has been reported to be 1% to 5% [16]. Recently, a large cohort study from the United Kingdom reported that any alcohol consumption is associated with risk [17].

Assuming that the Swedish National Board of Health and Welfare is successful in establishing a public acceptance for the new lower limit for risk-prone alcohol consumption and also enforces the provision of, as is discussed in their announcement [1], support to individuals at risk, the currently generally ignorant individual consuming risk-prone levels of alcohol will, to some extent, rethink and maybe accept the support provided. As the provision of simple, SMS text messaging–based support to people with risk-prone alcohol consumption has been shown to be moderately efficient [18], the support would probably be composed of different components. In addition to supporting behavioral change, an acute-phase test capable of indicating that alcohol has been recently consumed may be combined with a slower-phase test that indicates physiological status seen over the course of weeks or months. By regularly collecting data of different types with different temporal patterns, it is foreseeable that prediction algorithms can be developed to further support the individual at risk, similar to what is already available for alcohol use disorder [9].

One option for large-scale monitoring is to evaluate eye characteristics using mobile phones, similar to eye scanners developed for the management of substance use disorder [19]. These have been shown to detect benzodiazepine drugs, which act on the same receptor family as alcohol [20]. Under the assumption that the majority of the population owns a mobile phone, this test device is readily available in the target population.

Another option is saliva sampling to test for relevant molecular biomarkers. Saliva sampling is a noninvasive and user-friendly technique for the collection of biological samples that is also cost-efficient, particularly if provided as a home kit. A well-known example is the rapid diagnostic kits developed during the COVID-19 pandemic, which enabled home testing and infection monitoring using noninvasive sampling methods such as saliva [21]. Human saliva contains proteins and other components that are present in serum, and in certain instances, the concentration levels in both matrices are comparable [22,23]. From an alcohol monitoring perspective, the detection of biomarkers in saliva, for example, through rapid home tests, represents an advantageous approach for the early identification of drinking. Importantly, at-home testing offers an additional benefit of discretion, making it particularly suitable for individuals who prefer to keep their alcohol consumption private. However, there remains a need for saliva-based biomarkers that can reliably capture average alcohol consumption over 1 to 4 weeks, similar to the blood test phosphatidylethanol [24,25], and that can be implemented in rapid, user-friendly home tests.

In this project, we hypothesized that the physiological effects of moderate alcohol consumption can be measured by a simple analysis of relevant digital and molecular biomarkers. Such biomarkers are already well-established tools for diagnosis, prognosis, and monitoring of health conditions. We report a small pilot clinical study where the ability to detect alcohol consumption through secondary markers is evaluated in an exploratory manner. Only noninvasive tests that could be converted for large-scale home use were included in this small pilot clinical study to comply with the efficiency requirement. One acute-type test and 1 test with a slower timeline were studied. The evaluation of eye scanning was conducted using a platform that can readily be deployed at scale. In lieu of a suitable rapid home-use saliva test, levels of serotonin and orexin in saliva were quantified in a regular laboratory. The main goal of the study was to confirm the feasibility of the methods and collect preparatory information for a larger study.

## Methods

### Ethical Considerations

The study was approved by the ethics board (Etikprövningsmyndigheten; 2024-00815-01-521250) on February 5, 2024. All participants signed an informed consent form before enrolling in the study. Collected data were deidentified and linked only to a numeric participant identifier. The key for connecting numeric participant identifiers to actual personal identifiers was archived separately, only accessible by the primary investigator. No compensation was provided for participation. The study protocol has been deposited on Research Gate [26]. The first participant enrolled and signed the informed consent on September 9, 2024.

### Study Description

The study had the following inclusion criteria: individuals who were fully informed about the nature, scope, and relevance of the proposed exploratory study; individuals who voluntarily agreed to participate in the study and provided written informed consent; individuals who were aged between 18 and 67 years; women of childbearing age (18‐50 years) with a negative pregnancy test (via a urine test); and healthy individuals, without anamnestic data of relevant chronic disorders or regular use of medication for medical conditions.

Exclusion criteria [26] included blindness; individuals who had undergone eye surgery that could influence pupillary reflexes based on the research physician’s judgment; deafness; use of central depressants, sleeping pills, or medications contraindicating alcohol consumption; history or hereditary disposition for risk-prone use or dependency on alcohol; and regular alcohol consumption at or above the current recommendations for at-risk drinking.

The cohort consisted of 10 healthy volunteers recruited at a university campus in Sweden. The cohort comprised 4 (40%) male and 6 (60%) female participants aged 23 to 56 (median 45.4) years and weighing 60 to 125 (median 78.8) kg. In accordance with the study protocol [26], upon inclusion at visit 1, the participants received a brief training on how to use the eye-scanning app (version 4.3; Previct Drugs, Kontigo Care), and a saliva sample was donated (Salimetrics LLC). Visit 1 was held on several occasions to simplify recruitment. Between visit 1 and visit 2, the participants were asked to perform 12 eye-scanning tests in a sober condition because it is known that it may take some time before one learns how to conduct accurate eye-scanning events [20]. Visit 2 was the intervention when alcohol was consumed, taking place on 1 occasion when all participants were present. A total of 2 (20%) participants could not participate in visit 2 due to scheduling conflicts and were excluded from analysis. Among the 8 participants who were physically present during visit 2, a total of 5 (62.5%) completed all 12 eye-scanning tasks in the sober condition, whereas 3 (37.5%) completed between 5 and 11 tasks while sober. At visit 2, the participants first donated a saliva sample and performed an eye-scanning test and a breathalyzer test (Previct Alcohol, Kontigo Care) in a sober condition. Participants then repeated the procedure, consuming 1 glass of wine (white or red, 10%‐12% alcohol content, approximately 150 ml), waiting 15 minutes, completing an eye-scanning test, and subsequently performing a breathalyzer test, until either their blood alcohol concentration exceeded 0.6 per mille or they chose to discontinue. At the moment drinking stopped, a saliva sample was donated, and a usability questionnaire was completed. Subsequently, eye-scanning and breathalyzer measurements were recorded every 30 to 40 minutes for 1.5 to 3 hours. Before closing visit 2, a final saliva sample was donated.

### Data Collection and Analysis

The Previct Drugs system conducts eye scanning in a smartphone app with rigorous embedded quality control. This means that data of poor quality are prevented from being reported by the app in the smartphone. Consequently, the app may entirely disqualify an eye-scanning test. The collected data used for analysis are identical to the data that passed embedded quality control. Eye-scanning results were available as 13 key features, each representing a magnitude related to the pupillary light reflex, nystagmus, and the ability to converge the eyes, as previously described [19] and summarized in Table 1.

**Table 1.** Description of key features for the 3 test procedures—nonconvergence, nystagmus, and pupillary light reflex.

Key feature name	Description
Nonconvergence	
NCDiff	Ability to cross eyes
Nystagmus	
PeakCount	Number of nystagmus-like events
MotionMass	Magnitude of nystagmus-like events
Pupillary light reflex	
Dbase	Pupil size at ambient light
Dcon	Contracted pupil size after illumination
Dend	Pupil size at the end of measurement
Ctime	Time to maximum pupil contraction
MCA (Dbase-Dcon)	Maximum contraction amplitude
RMCA (Dbase-Dcon)/Dbase	Relative maximum contraction amplitude
MCV	Maximum contraction velocity
Latency	Time to first pupil reaction of illumination
PESC	Pupil escape (Dend-Dcon)
LeftEyeRedness	Color of sclera, left eye, expressed as CIELAB A

Saliva samples were examined for concentrations of serotonin and orexin using enzyme-linked immunosorbent assay (Invitrogen; catalog numbers EEL006 and EEL039). The change in measured concentration following alcohol consumption was evaluated by comparing the average value in a sober condition with results obtained at the highest blood alcohol concentration (using a 2-tailed *t* test) across all participants.

### Statistical Analysis

The sample size for this study was determined based on a recent study on eye scanning of people under the influence of drugs [19]. The impairment of eye characteristics due to alcohol consumption was evaluated by comparing the average key feature value of all data collected in a sober condition with the 3 measurement results obtained at highest blood alcohol level (using a paired *t* test) for each participant, relying on a significance level of *P*<.05. The effect size was estimated using Cohen *d*. The saliva sample results were evaluated by comparing average values of results obtained in a sober condition with average values of results obtained under the influence of alcohol (using a paired *t* test).

## Results

**Figure 1.**
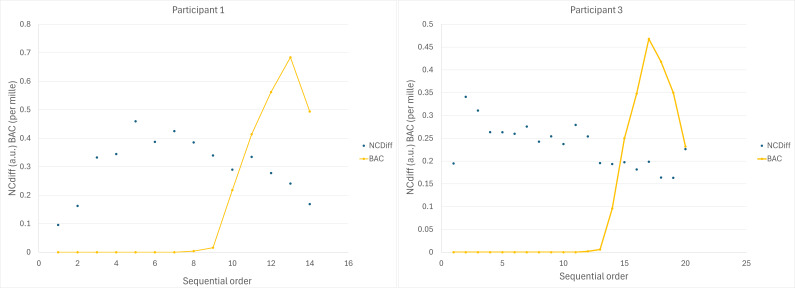
Time series of NCdiff (ability to cross eyes, expressed in arbitrary units [a.u.]) and blood alcohol concentration (BAC, expressed as per mille blood alcohol) for 2 participants. Participant 1 recorded 8 sober measurements, and participant 3 recorded 12 sober measurements before alcohol intake.

Eye-scanning results are presented in Table 2. Participant 9 was unable to complete any tests after having consumed alcohol. Effect sizes for significant (*P*<.05) observations were in the range of 0.50 to 2.5 (median 1.66, IQR 1.19-1.88). The ability to cross eyes, reported as the key feature NCdiff, often required some training. Time-series data are shown in Figure 1 for 2 participants. Participant 1 had difficulty crossing eyes in the beginning, but after 5 attempts, the NCdiff value was in the range of 0.4 to 0.5, which corresponds to clear ability to cross eyes. Upon consuming alcohol (measurement 9 onward), a gradual decline of convergence ability is seen. Participant 3 could cross eyes from start and lost some of the ability after having consumed alcohol (measurement 13 onward).

**Table 2.** *t* test results (*P* values) from the comparison of eye-scanning key feature values obtained in a sober condition with those obtained under the influence of alcohol.

Key feature values	Participant 1, *P* value	Participant 3, *P* value	Participant 4, *P* value	Participant 5, *P* value	Participant 6, *P* value	Participant 7, *P* value	Participant 8, *P* value
NCDiff	.33	.002	.051	.003	.86	.66	.78
PeakCount	.38	.10	.12	.97	.04	.91	.72
MotionMass	.94	.04	.19	.92	.06	.72	.84
Dbase	.19	.45	.32	.99	.65	.11	.62
Dcon	.03	.99	.63	.98	.93	.007	.72
Dend	.02	.48	.59	.03	.46	.07	.90
Ctime	.07	.03	.01	.51	.23	.49	.06
MCA	.33	.40	.24	.99	.49	.32	.58
RMCA	.44	.46	.23	.91	.38	.49	.66
MCV	.40	.82	.87	.60	.24	.08	.87
Latency	.76	.04	.72	.80	.39	.94	.59
PESC	.60	.52	.86	.02	.10	.09	.63
LeftEyeRedness	.47	.77	.98	.52	.15	.38	.21
Smallest *P* value per participant	.02	.002	.01	.003	.04	.007	.06

The usability of the eye-scanning app was generally good. In a sober condition, all participants managed to produce data of sufficient quality. When under the influence of alcohol at a blood alcohol concentration of approximately 0.5 per mille, the usability aspect changed. One participant was unable to produce any measurement with acceptable quality. Several participants failed 1 of the 6 eye-scanning tests conducted under the influence of alcohol. Overall, most participants exhibited reduced focus and diminished coordination at a blood alcohol concentration of 0.5 permille, consistent with expectations.

Participants 1, 3, and 9 deviated from the protocol and made too few sober tests at home before visit 2. No other protocol deviations were recorded.

One participant (participant 8) appeared to present an outlier pattern for breathalyzer measurements. According to the instructions for use of the breathalyzer, residual alcohol in the oral cavity can yield artificially elevated breathalyzer values. Hence, the protocol required a 15-minute waiting time between finishing a glass of wine and breathalyzer measurement. For participant 8, the following sequence was recorded: 1 glass of wine, blood alcohol concentration 0.06 per mille; second glass of wine, 0.2 per mille; third glass of wine, 0.66 per mille; and after a 30-minute wait, 0.22 per mille. No other participant in the study exhibited such a steep increase in blood alcohol concentration after a glass of wine, nor experienced such a rapid decrease over the following 30 minutes. Participant 8 was excluded from saliva biomarker analysis. Eye-scanning results are presented per participant, meaning that data from participant 8 should be evaluated with caution.

The 2 individuals weighing more than 100 kg both consumed 6 glasses of wine. Remaining participants consumed 3 to 4 glasses of wine, except for participant 8, who only had 1 glass of wine. One participant decided to stop drinking at 0.47 per mille. All other participants continued drinking until their blood alcohol concentration exceeded 0.6 per mille, with the highest recorded blood alcohol concentration being 0.72 per mille. Participants were, as anticipated, affected by alcohol consumption but only in a mild and transient manner. No serious adverse events were recorded.

When comparing averages across 7 participants, no significant differences were detected between saliva serotonin and orexin levels between the sober condition and after alcohol consumption (*P*=.50 and *P*=.40, respectively).

## Discussion

We report a small pilot clinical study of methods suitable for managing a large population with risk-level alcohol consumption. The small pilot study was also designed to produce procedural insights for a larger future study.

To ensure population-wide accessibility, we evaluated an eye-scanning tool integrated into a standard mobile phone, assuming that widespread mobile phone ownership makes the device readily available to a larger population. Eye-scanning results after alcohol consumption are in line with benzodiazepine impairment, which is logical because both benzodiazepines and alcohol interact with the gamma-aminobutyric acid receptor family [20]. In this study, we examined whether eye characteristics are affected after moderate alcohol intake. One identified visible impairment in eye movements, the loss of the ability to converge their eyes, appeared as an acquired skill, meaning that participants initially had to learn how to converge their eyes. Our tests indicated that while participants could converge their eyes well in the sober condition, they lost some ability to converge their eyes when under the influence of alcohol, and for some individuals, signs of eye impairment were already observable at blood alcohol concentrations of approximately 0.4 to 0.5 per mille. While the effects of alcohol on eye characteristics have been described for high levels of alcohol consumption (blood alcohol concentration around 0.8 per mille) [27], this study indicates that eye-related effects may also be observed at lower blood alcohol concentrations.

The importance of compliance is evident in this study. Of the 3 participants who did not complete all 12 sober baseline measurements, 1 was unable to produce any data under the influence of alcohol due to clumsiness. For the 2 participants with incomplete baseline data (participants 1 and 3), there is a risk that they had not fully learned how to converge their eyes before eye scanning under the influence of alcohol. This would result in a false negative result because both an inability to converge eyes and the loss of convergence due to intoxication will result in low NCdiff scores. We note that the operational procedures of Previct Drugs require 10 to 12 sober initial measurements for substance use disorder therapy. As Previct Drugs is deployed and used in the clinic, it is possible to conduct such sober baseline measurements for patients with a substance use disorder. Lack of compliance in this study was probably due to a lack of motivation.

The measurement of a bodily reaction to a drug for the purpose of indicating ingestion has many advantages and disadvantages. The test specificity will be vague because similar drugs will cause similar bodily reactions. The loss of convergence induced by both alcohol and benzodiazepines is one such example. Tests relying on altered bodily reactions will hence serve as screening tools in cases where confirmed use of a particular substance is desired. In other instances, such as estimating if a person is fit to operate a dangerous machine, the measurement of bodily reactions as such may be sufficient to allow access because the cause of the altered bodily reaction is irrelevant. The same reasoning can be used for designer drugs, where the development of specific chemical tests is slow. A broad test for the designer drug family, potentially relying on analysis of bodily reactions, could be sufficient to indicate the use of a designer drug, although it may not identify the specific substance. Finally, bodily reactions may be altered by conditions such as fatigue or arousal. All these aspects need to be taken into consideration when implementing a test based on measurements of bodily reactions.

Saliva sampling is a noninvasive method well suited for home use. However, it typically requires samples to be sent to a laboratory, which introduces logistical challenges and increases costs. Lateral flow rapid tests offer a cost-effective alternative by reducing the need for laboratory analysis, as was successfully demonstrated during the COVID-19 pandemic. Currently, no rapid tests are available for biomarkers such as serotonin or orexin, but they can be developed if required. Although the biomarkers evaluated in this pilot study did not yield reliable results, likely due to the small sample size, saliva sampling and analysis should not be dismissed. The primary advantage of saliva sampling is likely its ability to monitor the longer-term effects of alcohol consumption, which may not be captured in an acute-phase study such as the one reported here. With a larger study population and longer follow-up times, the identification of more suitable biomarkers, and continued development of rapid testing methods, this approach may still hold significant potential for future use.

Conducting a small pilot clinical study as a preparatory step for a larger study warranted several vital learnings. The acquired skill of converging eyes means that strict compliance with home measurements is important, and more than 12, preferably around 20, measurements at home are advisable to ensure that all individuals learn how to cross their eyes before the intervention. This would further allow statistical comparisons between “sober data after having acquired the skill of converging the eyes” and “convergence ability under the influence.” Saliva sampling was tested only on site in the pilot study, but it should also be tested at home if included in the next phase. Having 8 participants consume alcohol simultaneously during visit 2 proved to be challenging; therefore, reducing the number of participants to a maximum of 6 is advisable. Serving a stronger alcoholic beverage at least once or twice, and adjusting the amount consumed according to the participant’s body weight, are recommended to rapidly reach a blood alcohol concentration of approximately 0.4 per mille, thereby reducing the duration of alcohol exposure. To understand the eye-scanning performance at blood alcohol concentration of 0.4 per mille, we estimate that the population size should be around 72, but not less than 36. This would also allow a fair estimate of the proportion of individuals who are unable to converge their eyes. Increasing the target blood alcohol concentration from 0.6 to 0.7 or 0.8 per mille will allow better analysis of the relationship between alcohol concentration and the onset of convergence loss. Finally, to avoid potential false breathalyzer readings, it is recommended to rinse the oral cavity with water before conducting a breathalyzer measurement.

Managing risk-level alcohol use across a population of millions presents a public health challenge. The burden extends beyond the health care system where traditional intervention strategies such as in-person screening, counseling, and laboratory testing are resource intensive and logistically impractical to scale. Moreover, because many risk-level users lack self-awareness, they often do not seek help proactively; therefore, early signs of harmful use may go unnoticed by professionals until consequences become severe. This underscores the need for proactive, knowledge-based prevention strategies, particularly targeting women of childbearing age to reduce the risk of fetal alcohol spectrum disorders, in line with Sweden’s national public health objectives concerning alcohol and substance-related harm. Developing scalable, cost-effective, noninvasive, and nonintrusive tools for continuous assessment and engagement holds great potential to strengthen preventive measures at the population level and meet the growing societal demand for effective and discreet alcohol risk management.

In conclusion, this study demonstrated that alcohol consumption clearly affected eye characteristics in many participants, whereas the selected saliva biomarkers showed no measurable difference, at least with the measurement technique used. Therefore, the evaluated eye-scanning method may serve as a promising component for large-scale monitoring and support of at-risk individuals. Further studies, building on this pilot work, are warranted to confirm and extend these findings.

## Abbreviations

CBT: cognitive behavioral therapy
MI: motivational interviewing
